# The mitochondrial genomes of *Culex tritaeniorhynchus* and *Culex pipiens pallens* (Diptera: Culicidae) and comparison analysis with two other *Culex* species

**DOI:** 10.1186/s13071-016-1694-z

**Published:** 2016-07-21

**Authors:** Qian-Chun Luo, You-Jin Hao, Fengxia Meng, Ting-Jing Li, Yi-Ran Ding, Ya-Qiong Hua, Bin Chen

**Affiliations:** Institute of Entomology and Molecular Biology, College of Life Sciences, Chongqing Normal University, Chongqing, 401331 People’s Republic of China; National Institute for Communicable Disease Control and Prevention, Chinese Center for Disease Control and Prevention, Beijing, 102206 People’s Republic of China

**Keywords:** *Culex tritaeniorhynchus*, *Culex pipiens pallens*, *Culex*, Mitochondrial genome, Characteristics, Phylogenetics

## Abstract

**Background:**

*Culex tritaeniorhynchus* and *Culex pipiens pallens* are the major vectors of the Japanese encephalitis virus and *Wuchereria bancrofti*, the causative agent of filariasis. The knowledge of mitochondrial genomes has been widely useful for the studies on molecular evolution, phylogenetics and population genetics.

**Methods:**

In this study, we sequenced and annotated the mitochondrial (mt) genomes of *Cx. tritaeniorhynchus* and *Cx. p. pallens*, and performed a comparative analysis including four known mt genomes of species of the subgenus *Culex* (*Culex*). The phylogenetic relationships of *Cx. tritaeniorhynchus*, *Cx. p. pallens* and four known *Culex* mt genome sequences were reconstructed by maximum likelihood based on concatenated protein-coding gene sequences.

**Results:**

*Culex tritaeniorhynchus* and *Cx. p. pallens* mt genomes are 14,844 bp and 15,617 bp long, both consists of 13 PCGs, 22 tRNAs, 2 rRNAs and 1 CR (not sequenced for *Cx. tritaeniorhynchus*). The initiation and termination codons of PCGs are ATN and TAA, respectively, except for *COI* starting with TCG, and *COI* and *COII* terminated with T. tRNAs have the typical clover-leaf secondary structures except for *trnS*^*(AGN)*^ that is lacking the DHU stem. *16S rRNA* and *12S rRNA* secondary structures were drawn for the first time for mosquito mt genomes. The control region of *Cx. p. pallens* mt genome is 747 bp long and with four tandem repeat structures. Phylogenetic analyses demonstrated that the mt genome of *Cx. tritaeniorhynchus* was significantly separated from the remaining five mt genomes of *Culex* spp. *Culex p. pipiens*, *Cx. p. pallens* and *Cx. p. quinquefasciatus* formed a monophyletic clade with *Cx. p. quinquefasciatus* linked in the middle of the clade, and *Cx. p. pallens* should have the same taxonomic level as *Culex p. pipiens* and *Cx. p. quinquefasciatus*.

**Conclusions:**

The mt genomes of *Cx. tritaeniorhynchus* and *Cx. p. pallens* share the same gene composition and order with those of two other *Culex* species. *Culex p. pallens* of the Pipiens complex should have the same taxonomic level as *Culex p. pipiens* and *Cx. p. quinquefasciatus* investigated. We enriched the *Culex* mt genome data and provided a reference basis for further *Culex* mt genome sequencing and analyses.

**Electronic supplementary material:**

The online version of this article (doi:10.1186/s13071-016-1694-z) contains supplementary material, which is available to authorized users.

## Background

Mitochondrion, also known as “power plant”, is the structure for energy production and the main site for aerobic respiration in eukaryote cells [1]. Along with the rapid spread of mosquito-borne diseases, the research on mitochondrial (mt) genomes is of increasing importance to both, basic research and mosquito control. In most insects the mt genome is a small circular molecule with a length of *c*.15 Kb, containing 13 protein-coding genes (PCGs), 2 ribosomal RNA genes (rRNAs), 22 transfer RNA genes (tRNAs) and 1 (A + T)-rich control region (CR) [2–4]. Due to the stable structure, coding content conservation, maternal inheritance, rapid evolution rate, no recombination and a high copy number, the mt genome has been widely used in the pattern analysis of molecular evolution, and in the studies on phylogeography, phylogenetics and population genetics [5–9].

*Culex*, the largest genus in the Culicidae, is worldwide distributed [10], and its many species are important vectors of mosquito-borne diseases, including epidemic encephalitis and lymphatic filariasis [11]. So far, there have only been two species of this genus with mt genome sequences available in the GenBank database, *Cx. p. pipiens* and *Cx. quinquefasciatus* [12]. *Culex p. pipiens* is a recognized vector of encephalitis viruses in North America, Rift Valley fever virus in Egypt, and transmits lymphatic filariasis and canine dogworm in Eastern Asia, and *Cx. p. quinquefasciatus* is an important vector of filariasis in the tropics [13, 14]. There are four mt genome sequences for *C. p. pipiens* from different populations (three of these completely identical) and two mt genome sequences for *Cx. p. quinquefasciatus* with nucleotide differences, reported in the GenBank.

*Culex tritaeniorhynchus*, an important vector of Japanese encephalitis virus, belongs to the Sitiens group of the subgenus *Culex* and has a wide distribution in China, Japan, Korea, south-east Asia, India and Pakistan [15]. In 2012, there were 67,900 individuals infected by Japanese encephalitis virus, which led to 20,400 deaths and 14,000–24,000 cases of neurological impairment in Asia and the Western Pacific [16]. *Culex p. pallens*, belonging to the Pipiens complex of the Pipiens group, is widely distributed in China, Korea and Japan, and has been considered to be the major vector of bancroftian filariasis [15]. The classification of the Pipiens species complex has long been debated [17].

In the present study, we sequenced the mt genomes of *Cx. tritaeniorhynchus* and *Cx. p. pallens* with PCR amplifications, analyzed their characteristics including the composition and biases of nucleotides, codon usage, tRNA and rRNA secondary structure, and predicted tandem repeats of the control region. In addition, we carried out a comparative analysis of the newly-sequenced mt genomes with two other reported earlier in the genus, reconstructed the phylogenetic relationships using 13 protein-coding genes of six mt genome sequences, and discussed the classification of the Pipiens group. This comprehensive study of the known mt genomes within the genus *Culex* establishes an information frame of *Culex* spp. mt genomes and an important basis for further work.

## Methods

### Sample origin and mtDNA extraction

The colonies of *Cx. tritaeniorhynchus* and *Cx. p. pallens* were originally collected from fields in Beijing and Shaanxi, respectively, and reared at the National Institute for Communicable Disease Control and Prevention, Chinese Center for Disease Control and Prevention, Beijing. The adult samples were collected from the colonies, and then preserved in 100 % ethanol and stored at -80 °C prior to DNA extraction. Mitochondrial genomic DNA was extracted from single adult mosquito based on the method established in the Institute of Entomology and Molecular Biology, Chongqing Normal University, China [18].

### PCR amplification, sequencing, assembly and annotation

The complete mt genomes were amplified using 18 PCR primer pairs designed in Chongqing Normal University [19]. PCR amplification conditions included an initial denaturation at 94 °C for 1 min, followed by 32–36 cycles of denaturation at 94 °C for 40 s, annealing at 46–54 °C for 45 s, extension at 68 °C for 1 min and a final extension at 72 °C for 10 min. PCR products were column-purified (Qiagen QIAquick PCR Purification Kit, Hilden, Germany), separated by 1.0 % agarose gel electrophoresis, and sequenced with the same primers. The amplified fragments with low concentration or unclear electropherograms were cloned into the vector pMD-19 T (TaKaRa) according to the manufacturer’s recommendations before sequencing.

Mt genome sequences of *Cx. tritaeniorhynchus* and *Cx. p. pallens* were assembled into contigs using DNAMANx software. Annotations of the mt genomes were based on comparisons with mtDNA genes of *Cx. p. pipiens* and *Cx. quinquefasciatus*. Furthermore, the identification of the tRNA genes and drawing of the stem-loop secondary structures were conducted using the program tRNAscan-SE Search Server v.1.21 [20]. The identification of tRNA genes also referred to those in other two *Culex* spp. if the genes could not be completely recognized. rRNA genes were recognized by comparison with other mosquito sequences using comparative RNA Web (CRW) [21], and the secondary structures of rRNA were constructed with Mfold Web Server [22]. Nucleotide composition and codon usage were calculated using the programme MEGA 5.0 [23]. The six mt genome sequences were aligned using DNAMANx software, and sliding window analysis was performed using DnaSP V.5.10.01 [24], to reveal the nucleotide diversity at different nucleotide positions throughout the entire mt genomes. Each gene or unit skew analysis were assessed with the formulas: AT ‐ skew = (A %–T %)/(A % + T %) and GC ‐ skew = (G %–C %)/(G % + C %) [25]. In PCG genes, relative synonymous codon usage (RSCU) was calculated using the formula: the number of same codon coding a given amino acid divided by the total numbers of all synonymous codons coding the amino acid. The tandem repeat (TR) sequences in CR were examined using Tandem Repeats Finder program [26].

### Phylogenetic analysis

The phylogenetic relationships of the mt genome sequences for *Cx. tritaeniorhynchus*, *Cx. p. pallens* and four other known sequences for *Culex* spp. were reconstructed with *Anopheles gambiae* as the outgroup (Additional file 1: Table S1). The concatenated nucleotide sequences of 13 PCGs were used for the phylogenetic construction using Maximum Likelihood (ML) with PHYML [27]. The best-fit model of nucleotide substitution, the GTR + I + G model, was determined for the ML tree inference with Modeltest 3.7 [28]. The bootstrap values for 1,000 replicates and the genetic distances of each clade were calculated with PHYML, and the values larger than 50 % and the genetic distances were marked on each node of the tree.

## Results and discussion

### Annotation and base composition of the mt genomes of *Cx. tritaeniorhynchus* and *Cx. p. pallens*

**Fig. 1:**
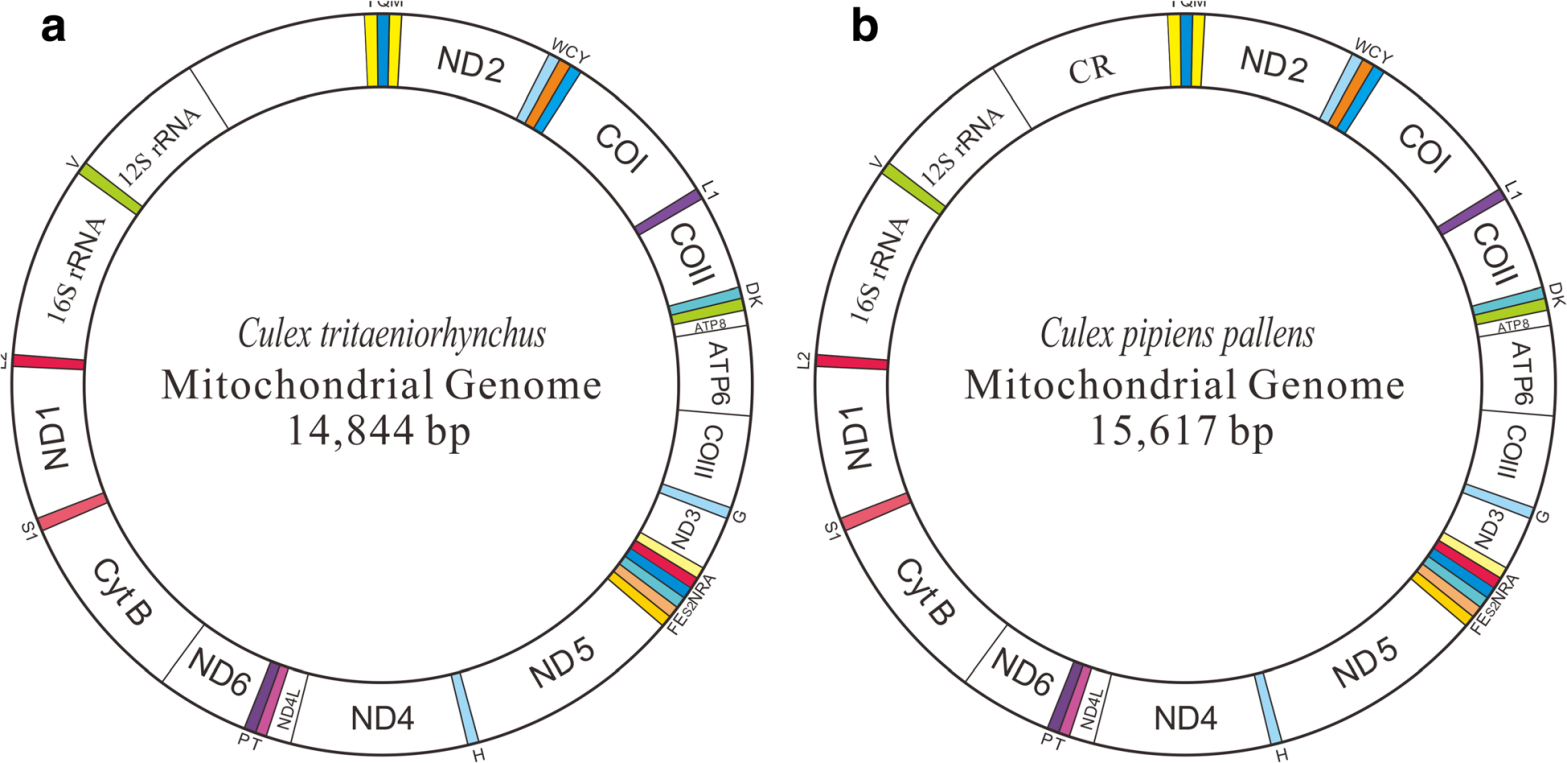
Structure of *Cx. tritaeniorhynchus* (**a**) and *Cx. p. pallens* (**b**) mt genome. The colour-filled blocks indicate tRNAs, and the white blocks indicate PCGs, rRNAs and control region (CR)

The circular mt genomes of *Cx. tritaeniorhynchus* and *Cx. p. pallens* were 14,844 bp (Fig. 1a, GenBank number KT851544; CR lacking) and 15,617 bp long (Fig. 1b, GenBank number KT851543, with CR), respectively. They both consisted of 13 PCGs, 22 tRNAs, 2 rRNAs and 1 CR (not sequenced for *Cx. tritaeniorhynchus*), which is a composition typical for the metazoan taxa [2] and conserved in the known mosquito mt genomes [29, 30]. There were 16 overlapping nucleotide areas between genes/CR with a total length of 46 bp and 74 bp, respectively; these areas ranged between 1–7 bp except for that between 12S rRNA and CR region of *Cx. p. pallens*, which was 34 bp long (Table 1). There were 12 intergenic spacers with a total length of 79 bp and 90 bp, respectively, in these two species, and the spacers ranged between 1–19 bp.

**Table 1:** Positions and features of the genes in *Cx. tritaeniorhynchus* and *Cx. p. pallens* mt genomes

Gene (strand)	Position in *Cx. p. pallens* (*Cx. tritaeniorhynchus*)	Size (bp)	Intergenic spacers (bp) number	Anti-codon	Start/stop-codon
*Ile* (H)	1–69	69		GAT	
*Gln* (L)	70–138	69	0	TTG	
*Met* (H)	142–210	69	3	CAT	
*ND2* (H)	211–1233	1023	0		ATC/TAA
*Trp* (H)	1238–1306	69	4	TCA	
*Cys* (L)	1306–1372	67	-1	GCA	
*Tyr* (L)	1385–1450 (1373–1437)	66 (65)	12 (0)	GTA	
*CO I* (H)	1449–2985 (1436–2972)	1537	-2		TCG/T
*Leu* (H)	2986–3052 (2973–3039)	67	0	TAA	
*CO II* (H)	3070–3754 (3046–3730)	685	17 (6)		ATA(ATG)/T
*Lys* (H)	3755–3826 (3731–3801)	72 (71)	0	CTT	
*Asp* (H)	3839–3906 (3811–3878)	68	12 (9)	GTC	
*ATP8* (H)	3916–4068 (3888–4040)	153	9		ATA/TAA
*ATP6* (H)	4062–4742 (4034–4714)	681	-7		ATG/TAA
*CO III* (H)	4742–5530 (4714–5502)	789	-1		ATG/TAA
*Gly* (H)	5530–5596 (5502–5568)	67	-1	TCC	
*ND3* (H)	5594–5950 (5566–5922)	357	-3		ATA/TAA
*Arg* (H)	5949–6012 (5921–5988)	64 (68)	-2	TCG	
*Ala* (H)	6013–6078 (6002–6067)	66	0 (13)	TGC	
*Asn* (H)	6079–6145 (6606–6134)	67	0	GTT	
*Ser* (L)	6148–6214 (6138–6204)	67	2 (3)	GCT	
*Glu* (H)	6216–6281 (6206–6274)	66 (69)	1	TTC	
*Phe* (L)	6280–6346 (6272–6339)	67 (68)	-2 (-3)	GAA	
*ND5* (L)	6347–8101 (6334–8079)	1755 (1746)	0 (-6)		ATC/TAA
*His* (L)	8099–8164 (8077–8142)	66	-3	GTG	
*ND4* (L)	8164–9507 (8142–9485)	1344	-1		ATG/TAA
*ND4L* (L)	9501–9800 (9475–9775)	300 (297)	-7		ATG/TAA
*Thr* (H)	9806–9870 (9781–9846)	65 (66)	5	TGT	
*Pro* (L)	9871–9936 (9847–9912)	66	0	TGG	
*ND6* (H)	9942–10457 (9918–10433)	516	5		ATA/TAA
*Cyt B* (H)	10457–11593 (10433–11572)	1137 (1140)	-1		ATG/TAA
*Ser* (H)	11593–11658 (11573–11638)	66	-1 (0)	TGA	
*ND1* (L)	11677–12633 (11658–12614)	957	18 (19)		ATA/TAA
*Leu* (L)	12628–12694 (12609–12676)	67 (68)	-6	TAG	
16S *rRNA* (L)	12697–14030 (12679–14016)	1334 (1338)	2		
*Val* (L)	14029–14100 (14015–14086)	72	-2	TAC	
12S *rRNA* (L)	14101–14904 (14087–14843)	804 (757)	0		
CR	14871–15617 (NO)	747	-34		

*Abbreviations*: *H* heavy strand, *L* light strand

**Fig. 2:**
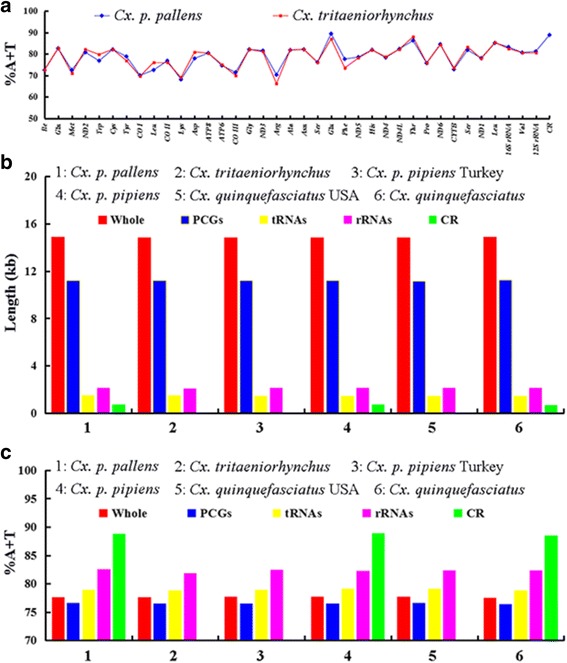
AT contents and length of genes or regions in the six *Culex* mt genomes compared. **a** AT percentage of each gene or region in *Cx. tritaeniorhynchus* and *Cx. p. pallens*. **b** and **c** Length (**b**) and AT percentage (**c**) of whole mt genome, PCGs, tRNAs, rRNAs and CR of the six mt genomes investigated

The mt genomes of *Cx. tritaeniorhynchus* showed a high nucleotide bias with 77.6 % of AT and 22.4 % of GC (39.1 % A; 38.5 % T; 9.4 % G; and 13.0 % C). The overall nucleotide composition of *Cx. p. pallens* was 39.5 % A, 38.7 % T, 9.1 % G and 12.7 % C, and CR had the highest AT content (88.8 %). The AT contents of rRNAs, tRNAs and PCGs in *Cx. tritaeniorhynchus* were 81.8 %, 78.8 % and 76.5 %, and *Cx. p. pallens* has similar values: 82.5 %, 78.9 % and 76.6 % (Fig. 2c). The AT content of 37 genes in the two mt genomes ranged from 66.2 % (*trnH*) to 89.4 % (*trnN*) (Fig. 2a), with nine genes (*trnI*, *trnQ*, *trnC*, *ATP8*, *trnG*, *trnA*, *trnN*, *trnS* and *trnV*) being identical and four genes (*trnF*, *trnR*, *trnD* and *trnL*) exhibiting the largest differences.

### Protein-coding genes

The total length of PCGs in the two mt genomes was 11,225 bp and 11,234 bp (Fig. 2b), with AT content 76.5 % (32.4 % A; 44.1 % T; 11.2 % C; and 12.3 % G) and 76.6 % (32.3 % A; 44.3 % T; 11.0 % C; and 12.4 % G) (Table 2), respectively. The AT content at the 1st, 2nd and 3rd codon position were all above 67.3 %, with that in the 3rd codon position reaching 92.4–92.9 %, which is a characteristic widespread in insect mt genomes [31]. The PCGs of six *Culex* mt genomes showed an overall negative AT-skew and positive GC-skew, with negative AT-skew at the 1st, 2nd and 3rd codon position except for the 3rd position of *Cx. p. pallens* mt genome, and positive GC-skew at the 1st, but negative at the 2nd and 3rd codon position (Table 2).

**Table 2:** Base compositions of 13 PCGs in six *Culex* spp. mt genomes

Species	% A	% T	% C	% G	% A + T	% C + G	AT-skew	GC-skew
*Cx. p. pallens*	32.3	44.3	11.0	12.4	76.6	23.4	-0.16	0.06
1st	31.8	37.8	10.7	19.6	69.6	30.3	-0.09	0.29
2nd	21.0	46.3	18.5	14.3	67.3	32.8	-0.38	−0.13
3rd	48.7	44.1	3.8	3.4	92.8	7.2	0.05	−0.06
*Cx. tritaeniorhynchus*	32.4	44.1	11.2	12.3	76.5	23.5	-0.15	0.05
1st	31.7	38.1	10.8	19.4	69.8	30.2	-0.09	0.28
2nd	21.2	46.1	18.5	14.2	67.3	32.7	-0.37	−0.13
3rd	44.3	48.1	4.2	3.3	92.4	7.5	-0.04	−0.12
*Cx. p. pipiens* (*Cx. p. pipiens* Turkey)	32.2 (32.2)	44.3 (44.4)	11.0 (11.0)	12.4 (12.4)	76.5 (76.6)	23.4 (23.3)	-0.16 (-0.16)	0.06 (0.06)
1st	32.4 (31.7)	38.0 (38.0)	10.6 (10.7)	19.1 (19.6)	70.4 (69.7)	29.7 (30.3)	-0.08 (-0.09)	0.29 (0.29)
2nd	20.5 (20.8)	46.8 (46.4)	18.6 (18.5)	14.0 (14.3)	67.3 (67.2)	32.6 (32.8)	-0.39 (-0.38)	−0.14 (−0.13)
3rd	44.3 (44.2)	48.5 (48.7)	3.7 (3.7)	3.5 (3.3)	92.8 (92.9)	7.2 (7.0)	-0.05 (-0.05)	−0.03 (−0.06)
*Cx. quinquefasciatus* (*Cx. quinquefasciatus* USA)	32.1 (32.2)	44.3 (44.4)	11.1 (11.0)	12.5 (12.4)	76.4 (76.6)	23.6 (23.3)	-0.16 (-0.16)	0.06 (0.06)
1st	32.8 (31.7)	38.3 (38.0)	10.3 (10.7)	18.6 (19.6)	71,1 (69.7)	28.9 (30.3)	-0.08 (-0.09)	0.29 (0.29)
2nd	21.6 (20.7)	46.1 (46.4)	17.7 (18.6)	14.6 (14.3)	67.7 (67.1)	32.3 (32.9)	-0.36 (-0.38)	−0.10 (−0.13)
3rd	42.0 (44.2)	48.4 (48.7)	5.4 (3.7)	4.2 (3.4)	90.4 (92.9)	9.6 (7.1)	-0.07 (-0.05)	−0.125 (−0.04)

The PCGs in *Cx. tritaeniorhynchus* used the initiation codon ATN except for *COI* that had the initiation codon TCG [8, 29, 32], and six genes (*ATP6, COIII, COII, Cytb, ND4* and *ND4L*), four genes (*ATP8, ND1, ND3* and *ND6*) and the other genes (*ND2* and *ND5*) used the initiation codon ATG, ATA and ATC, respectively (Table 3). *Culex p. pallens* mt genome had the same initiation codon except for *COII* that used ATA. These two mt genomes had the complete termination codon TAA except for *COI* and *COII* that had the incomplete termination codon T, which was supplied to complete termination codon TAA by polyadenylations during the posttranscriptional process [33]. A comparison of the six mt genomes revealed that termination codons were different in *COIII*, *CytB*, *ND3* and *ND4*.

**Table 3:** Start (T) and stop (P) codons of 13 PCGs in six *Culex* spp. mt genomes

		*ATP6*	*ATP8*	*COI*	*COII*	*COIII*	*Cytb*	*ND1*	*ND2*	*ND3*	*ND4*	*ND4L*	*ND5*	*ND6*
*Cx. p. pallens*	T	ATG	ATA	TCG	ATA	ATG	ATG	ATA	ATC	ATA	ATG	ATG	ATC	ATA
	P	TAA	TAA	T	T	TAA	TAA	TAA	TAA	TAA	TAA	TAA	TAA	TAA
*Cx. tritaeniorhynchus*	T	ATG	ATA	TCG	ATG	ATG	ATG	ATA	ATC	ATA	ATG	ATG	ATC	ATA
	P	TAA	TAA	T	T	TAA	TAA	TAA	TAA	TAA	TAA	TAA	TAA	TAA
*Cx. p. pipiens* Turkey	T	ATG	ATA	TCG	ATG	ATG	ATG	ATA	ATC	ATA	ATG	ATG	ATC	ATA
	P	TAA	TAA	T	T	TA	T	TAA	TAA	T	TA	TAA	TAA	TAA
*Cx. p. pipiens*	T	ATG	ATA	TCG	ATG	ATG	ATG	ATA	ATC	ATA	ATG	ATG	ATC	ATA
	P	TAA	TAA	T	T	TA	T	TAA	TAA	T	TA	TAA	TAA	TAA
*Cx. quinquefasciatus* USA	T	ATG	ATA	TCG	ATG	ATG	ATG	ATA	ATC	ATA	ATG	ATG	ATC	ATA
	P	TAA	TAA	T	T	TA	T	TAA	TAA	T	TA	TAA	TAA	TAA
*Cx. quinquefasciatus*	T	ATG	ATA	TCG	ATG	ATG	ATG	ATA	ATC	ATA	ATG	ATG	ATC	ATA
	P	TAA	TAA	T	T	TAA	TAA	TAA	TAA	T	TAA	TAA	TAA	TAA

**Fig. 3:**
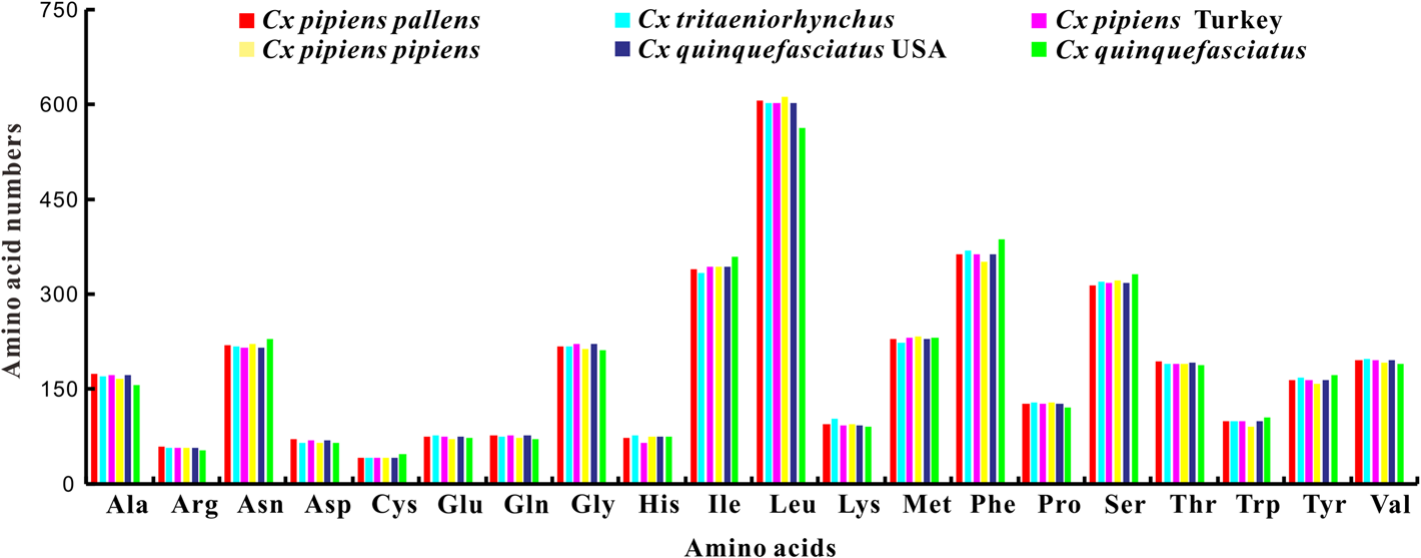
Amino acids and their numbers in the six *Culex* spp. mt genomes investigated

There were 58 and 60 different codons used in *Cx. tritaeniorhynchus* and *Cx. p. pallens* mt genomes, respectively (Table 4). RSCU analysis showed that UUA, CGU, GCU, UCU and GGA were most used codons in the two mt genomes, and GGC, UGG, ACG and UCG were least used in *Cx. tritaeniorhynchus*, whereas CUG, CUC, GGC and AGG were least used in *Cx. p. pallens*. Results of the RSCU analysis showed that the codons with A and T in the 3rd position were overused when compared to other synonymous codons. For example, the codon TTA for Leu presented a RSCU value of 5.08 and 5.14 in the two mt genomes, and the codon CTG also for Leu showed a RSCU value of 0 and 0.01, respectively. The Leu was most present amino acid; on the contrary, Cys was least used one in the amino acid sequences of the two mt genomes, which were consistent with other *Culex* mt genomes studied to date (Fig. 3).

**Table 4:** Relative synonymous codon usages (RSCU) in *Cx. tritaeniorhynchus* (CT) and *Cx. p. pallens* (CP) mt genomes. The abbreviations of the amino acids coded are shown in parentheses

Codon	RSCU		Codon	RSCU		Codon	RSCU		Codon	RSCU	
	CP	CT		CP	CT		CP	CT		CP	CT
UUU(F)	1.85	1.74	UCU(S)	2.64	2.63	UAU(Y)	1.82	1.83	UGU(C)	1.9	2
UUC(F)	0.15	0.26	UCC(S)	0.18	0.15	UAC(Y)	0.18	0.17	UGC(C)	0.1	0
UUA(L)	5.14	5.08	UCA(S)	2.08	2.25	UAA(*)	2	2	UGA(W)	1.92	1.98
UUG(L)	0.21	0.24	UCG(S)	0.13	0.03	UAG(*)	0	0	UGG(W)	0.08	0.02
CUU(L)	0.39	0.34	CCU(P)	2.32	2.26	CAU(H)	1.7	1.58	CGU(R)	0.47	0.7
CUC(L)	0.01	0	CCC(P)	0.22	0.34	CAC(H)	0.3	0.42	CGC(R)	0	0
CUA(L)	0.25	0.34	CCA(P)	1.46	1.4	CAA(Q)	1.92	1.92	CGA(R)	3.33	3.23
CUG(L)	0.01	0	CCG(P)	0	0	CAG(Q)	0.08	0.08	CGG(R)	0.2	0.07
AUU(I)	1.87	1.93	ACU(T)	2.06	2.08	AAU(N)	1.81	1.83	AGU(S)	1.78	1.63
AUC(I)	0.13	0.07	ACC(T)	0.08	0.13	AAC(N)	0.19	0.17	AGC(S)	0.1	0.15
AUA(M)	1.81	1.79	ACA(T)	1.86	1.77	AAA(K)	1.53	1.47	AGA(S)	1.04	1.1
AUG(M)	0.19	0.21	ACG(T)	0	0.02	AAG(K)	0.47	0.53	AGG(S)	0.05	0.07
GUU(V)	2.04	2.02	GCU(A)	2.74	2.8	GAU(D)	1.83	1.75	GGU(G)	0.84	0.79
GUC(V)	0.08	0.08	GCC(A)	0.18	0.21	GAC(D)	0.17	0.25	GGC(G)	0.06	0.02
GUA(V)	1.76	1.82	GCA(A)	0.92	0.92	GAA(E)	1.92	1.92	GGA(G)	2.55	2.69
GUG(V)	0.12	0.08	GCG(A)	0.16	0.07	GAG(E)	0.08	0.08	GGG(G)	0.55	0.5

*Stop codon

### Transfer RNA and ribosomal RNA genes

Out of 22 tRNAs in the two mt genomes, 18 were predicted using tRNAscan-SE Search Server v.1.21 [20], and the remaining four tRNAs (*trnS*, *trnR*, *trnK* and *trnL*) were identified by comparing with known *Culex* spp. mt genomes. The tRNAs of *Cx. tritaeniorhynchus* totaled 1,490 bp in length (Fig. 2b), had a 78.8 % AT content (Fig. 2c) and ranged from 65 bp (*trnY*) to 72 bp (*trnV*); those of *Cx. p. pallens* totaled 1,482 bp, had 78.9 % AT content and ranged from 64 bp (*trnR*) to 72 bp (*trnV*). The anticodons of the 22 tRNAs were identical with published reference mosquito mt genomes [29, 30] (Table 1). The tRNA secondary structure is a typical clover-leaf structure in many insects, including four stems [dihydorouridine (DHU), amino acids (AA), TΨC, anticodons (AC)] and loops [DHU, TΨC, AC and variable (V)] [34–36]. The 22 tRNAs of the *Cx. tritaeniorhynchus* and *Cx. p. pallens* mt genomes also had the typical clover-leaf secondary structures except for *trnS*^*(AGN)*^ that was lacking the DHU stem (Additional file 2: Figure S1a) and contained 30 and 25 mismatched base pairs, respectively, which were distributed in AA stem (8 and 6 bp), DHU stem (11 and 10 bp), AC stem (8 and 6 bp) and TΨC (3 bp in both species). Those mismatched base pairs affect the thermodynamic stability compared with Watson-Crick pairs [37, 38], and are a common phenomenon but could be corrected in posttranscriptional RNA editing processes [39].

The 12S rRNAs in the *Cx. tritaeniorhynchus* and *Cx. p. pallens* mt genomes were both located between *trnV* and CR and the 16S rRNAs between *trnL* and *trnV*, as in the mt genomes in other metazoan species [29, 30, 32]. The 12S rRNAs were 757 bp and 804 bp long with AT contents 80.6 % and 81.2 %, respectively, and the 16S rRNAs were 1,338 bp and 1,334 bp long with AT contents 82.5 % and 83.2 %, respectively. The rRNA secondary structures were inferred for the first time for a mosquito mt genome (Additional file 2: Figure S1b and c). They all contained G-C, A-U and G-U base pairs with G-U not being considered as mismatched base pairs [37]. The secondary structures contained terminal loops (T: 13 and 23), interior loops (I: 15 and 20) and helices (H: 24 and 42), as in mt genomes in other insects.

### The control regions

**Fig. 4:**
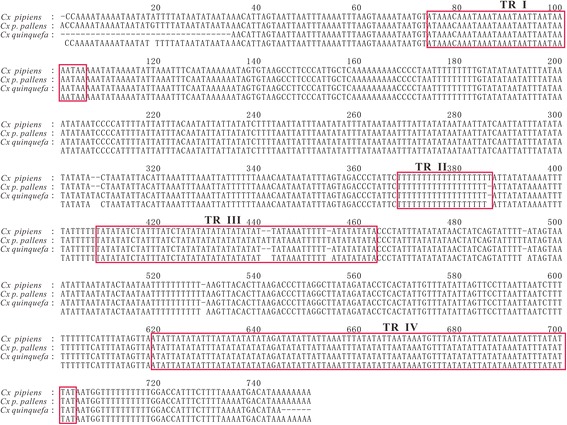
Alignment and predicted tandem repeat (TR) structures in the mt genomes of *Cx. p. pallens*, *Cx. pipiens* and *Cx. quinquefasciatus*

The CR of *Cx. p. pallens* mt genome was 747 bp long, located between *12S rRNA* and *trnI* (Table 1), and had a highest AT content 88.8 %. We identified four TR structures (Fig. 4): TR I (nucleotides 14,966–14,998 in the mt genome sequence), TR II (15,258–15,275), TR III (15,297–15,335) and TR IV (15,508–15,593). The TR I was 33 bp long and composed of an ATAA unit, TR II 18 bp and poly-T, TR III 39 bp and microsatellites (TA)_n_ and TR IV 86 bp, and microsatellites (AT)_n_. The comparison with two other known *Culex* spp. mt genomes revealed that these four TR structures were highly conserved, with exception of a thymine change in TR II and two thymine and one adenine changes in TR III in *Cx. p. pallens* (Fig. 4).

### Nucleotide diversity throughout the whole mt genome

**Fig. 5:**
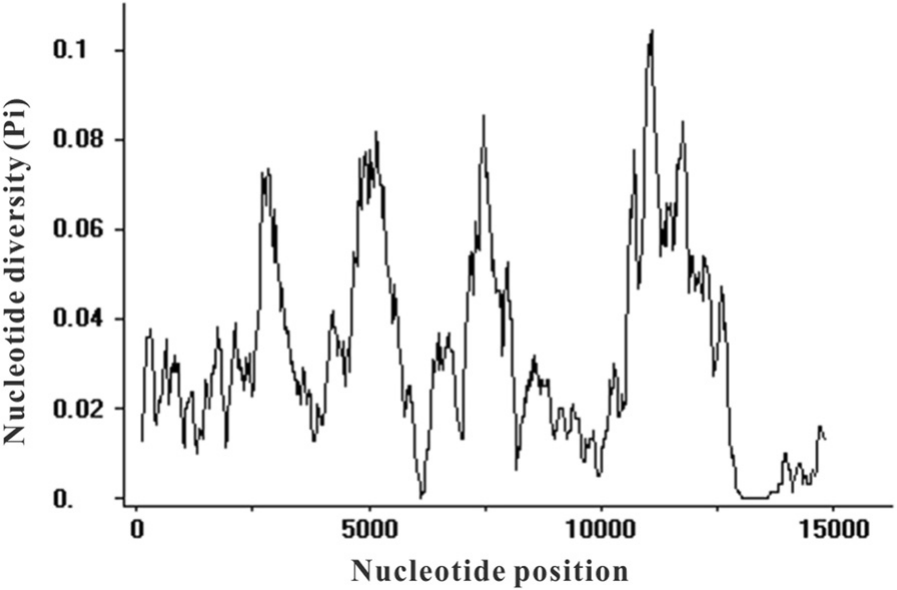
Nucleotide diversity of the six *Culex* spp. mt genomes produced by sliding window analysis. Windows of 200 bp with step size of 25 bp were applied in the analysis

The aligned sequence length of the six *Culex* spp. mt genomes was 15,669 bp, and the sliding window analysis showed that nucleotide diversity (*Pi*) value at nucleotide positions ranged from 0 to 0.10433 (Fig. 5). There were four high nucleotide divergence regions identified with the *Pi*-values greater than 0.06 in each region, and these regions were located at 2,592–3,079 bp, 4,684–5,437 bp, 7,192–7,700 bp and 10,511–13,701 bp, respectively. In comparisons of different types of genes, protein-coding genes had higher *Pi*-values, with the highest *Pi*-value found in *ND1* (*Pi* = 0.02733–0.10433), followed by *ND5* (*Pi* = 0.01667–0.08533), *CytB* (*Pi* = 0.02000–0.08233), *COIII* (*Pi* = 0.03900–0.08200), *ATP6* (*Pi* = 0.02333–0.07600), *COI* (*Pi* = 0.02333–0.07167) and others (*Pi* ≤ 0.06, so not in the four high nucleotide divergence regions). Out of the 22 tRNAs, only two tRNAs [*trnL*^(*UUR*)^ (*Pi* = 0.04167–0.06433) and *trnS*^(*UCN*)^ (*Pi* = 0.06300–0.07833)] had *Pi* ≥ 0.06, and were located in the high nucleotide divergence regions at 2,985–3,051 and 11,592–11,657, respectively. Two rRNA genes (12S rRNA and 16S rRNA) were the most conserved with *Pi* = 0. Based on the nucleotide diversity, the protein-coding genes would be most suitable as markers to elucidate the phylogenetic relationships at the genus level.

### Phylogenetic analysis

**Fig. 6:**
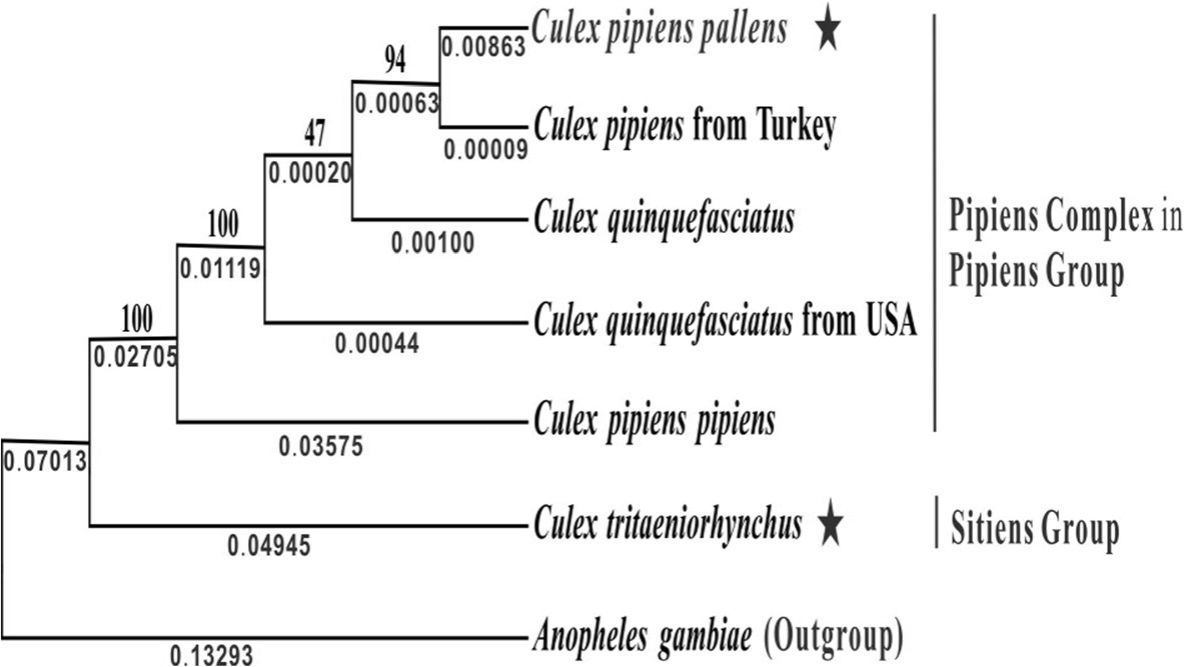
Phylogenetic tree of the six *Culex* spp. mt genomes based on nucleotide sequences of 13 protein-coding genes. Maximum Likelihood analysis was used to construct the tree with *Anopheles gambiae* as the outgroup. The genetic distances and bootstrap values from 1,000 replicates are marked at the nodes. Stars indicate the newly-generated mt genome sequences

Due to the high conservation of the amino acid sequences in the six *Culex* spp. mt genomes with close phylogenetic relationships, the ML method was used to infer the phylogenetic relationships using nucleotide sequences of 13 PCGs with *An. gambiae* as the outgroup (Fig. 6). On the tree, the mt genome of *Cx. tritaeniorhynchus* appeared separate from the remaining five which formed a strongly supported clade (100 % bootstrap support and a genetic distance of 0.02705). This result is consistent with traditional classification, in which *Cx. tritaeniorhynchus* is assigned to the Vishnui Subgroup in the Sitiens Group of the subgenus *Culex*, and the remaining species to the Pipiens complex of the Pipiens Subgroup in the Pipiens Group of the same subgenus [13]. *Culex p. pallens* and *Cx. pipiens* from Turkey were grouped with 94 % bootstrap support and a genetic distance of 0.00063. Interestingly, the two mt genomes of *Cx. quinquefasciatus* were linked between *Cx. p. pallens* and *Cx. p. pipiens*, and their separation was not supported (47 % bootstrap support), with a very small genetic distance of 0.00020. *Culex p. pipiens* was separated from the other four mt genomes in the Pipiens complex with 100 % of bootstrap support and a relatively large genetic distance of 0.01119.

Miller et al. [40] constructed a phylogeny of the Pipiens species complex based on rDNA ITS-1 and ITS-2 (1,326 aligned sites). In the tree involving 14 *Culex* spp., *Cx. p. pipiens, Cx. p. pallens* and *Cx. quinquefasciatus* were clustered into a unique clade, inside which there was seldom further divergence. In the tree involving 26 mosquito species representing 13 geographical populations of the Pipiens complex, there were two large clades: the *Cx. quinquefasciatus* clade and *Cx. pipiens* clade, and the intermediate pipiens-quinquefasciatus hybrids formed a group with *Cx. pipiens pallens*, which was linked to the *Cx. pipiens* clade. The hybridization in the Pipiens complex has been widely reported, e.g. between *Cx. p. pipiens* and *Cx. p. quinquefasciatus* in Madagascar [41], Argentina [42] and California [43], and between *Cx. p. pallens* and *Cx. quinquefasciatus* in southern Japan, Korea and China [44, 45]. More recently, Liu et al. [14] investigated genetic polymorphisms in the Pipiens Complex in Lhasa, China, using multiplex PCR and sequencing at the 2nd intron of *ace-2*. The results revealed that 36 mosquitoes (34.29 % of the total) were homozygous (13 *Cx. p. pipiens*, 20 *Cx. p. pallens* and 3 *Cx. p. quinquefasciatus*), whereas 69 (65.71 %) were heterozygous (41 between *Cx. p. pipiens* and *Cx. p. pallens*, 1 *Cx. p. pipiens* and *Cx. p. quinquefasciatus*, 14 *Cx. p. pallens* and *Cx. p. quinquefasciatus*, and 13 among *Cx. p. pipiens*, *Cx. p. pallens* and *Cx. p. quinquefasciatus*). These results demonstrated that the three “subspecies or species” can cross each other in sympatry.

For the Pipiens complex, Harbach [17] used the Pipiens Assemblage to avoid difficulties associated with the meaning of the word “complex”. He concluded that *Cx. pipiens* and *Cx. quinquefasciatus* were separate species which evolved in Africa and hybridize in non-indigenous areas where they were unintentionally introduced by humans; and *Cx. pallens* has no taxonomic status under the provisions of the International Code of Zoological Nomenclature.

Whether the Pipiens Assemblage is a single polytypic species or a complex of sibling species has long been disputed [17]. Despite extensive morphological and physiological/behavioral variation, there has been no conclusive phylogenetic analysis to support their species status and the hybridization widely occurs where their populations overlap. Our study based on mt genomes strongly suggests that *Cx. p. pipiens, Cx. p. pallens* and *Cx. p. quinquefasciatus* were monophyletic, but their taxonomic status is still unsettled. Considering the bootstrap support and genetic distances in the present analysis, these all should have same taxonomic level, species, subspecies or form.

## Conclusions

We sequenced and analyzed the mt genomes of *Cx. tritaeniorhynchus* and *Cx. p. pallens*. The gene composition and order of the two mt genomes are the same as in the six known mt genomes of two *Culex* spp. The rRNA secondary structures were described for the first time for a mosquito mt genome. *Culex tritaeniorhynchus* was separated from the remaining five *Culex* spp. mt genomes, consistent with the traditional classification, in which *Cx. tritaeniorhynchus* is assigned to the Sitiens Group and the remaining species to the Pipiens complex in the Pipiens Group. *Culex p. pipiens*, *Cx. p. pallens* and *Cx. p. quinquefasciatus* were monophyletic, indicating that they should be recognised at the same taxonomic level although their taxonomic status is still unsettled.

## Abbreviations

bp, base pair; CR, control region; CRW, comparative RNA web; ML, maximum likelihood; Mt genome, mitochondrial genome; PCGs, protein-coding genes; *Pi*, nucleotide diversity; rRNAs, ribosomal RNA genes; RSCU, relative synonymous codon usage; TR, tandem repeats; tRNAs, transfer RNA genes

## Additional files

Additional file 1: Table S1. GenBank accession numbers for the mt genomes of the mosquito species used in this study. (PDF 21 kb) Additional file 2: Figure S1. Predicted secondary structure for 22 tRNAs (**a**), 12S rRNA (**b**) and 16S rRNA (**c**) in *Cx pipiens pallens* and *Cx tritaeniorhynchus* mt genomes. (PDF 240 kb)

## References

[1] Wei SJ, Chen XX (2011). Progress in research on the comparative mitogenomics of insects. Chin J Appl Entomol.

[2] Boore JL (1999). Animal mitochondrial genomes. Nucleic Acids Res.

[3] Goddard JM, Wolstenholme DR (1980). Origin and direction of replication in mitochondrial DNA molecules from the genus *Drosophila*. Nucleic Acids Res.

[4] Simon C, Buckley TR, Frati F, Stewart JB, Beckenbach AT (2006). Incorporating molecular evolution into phylogenetic analysis, and a new compilation of conserved polymerase chain reaction primers for animal mitochondrial DNA. Ann Rev Ecol Evol Syst.

[5] Wilson K, Cahill V, Ballment E, Benzie J (2000). The complete sequence of the mitochondrial genome of the crustacean *Penaeus mondon*: are malacostracan crustaceans more closely related to insects than to branchiopods?. Mol Biol Evol.

[6] Brown WM, George M, Wilson AC (1979). Rapid evolution of animal mitochondrial DNA. Proc Natl Acad Sci U S A.

[7] Krzywinski J, Besansky NJ (2003). Molecular systematics of *Anopheles*: from subgenera to subpopulations. Annu Rev Entomol.

[8] Krzywinski J, Grushko OG, Besansky NJ (2006). Analysis of the complete mitochondrial DNA from *Anopheles funestus*: an improved dipteran mitochondrial genome annotation and a temporal dimension of mosquito evolution. Mol Phylogenet Evol.

[9] Krzywinski J, Wilkerson RC, Besansky NJ (2001). Evolution of mitochondrial and ribosomal gene sequences in Anophelinae (Diptera: Culicidae): implications for phylogeny reconstruction. Mol Phylogenet Evol.

[10] Harbach RE. *Culex* classification. Mosquito Taxonomic Inv. 2013. http://mosquito-taxonomic-inventory.info/ltemgtculexltemgt-classification.

[11] Lu BL (1997). Fauna Sinica. Insecta. Diptera: Culicidae 1. Vol. 8.

[12] Behura SK, Lobo NF, Haas B, de Bruyn B, Lovin DD, Shumway MF (2011). Complete sequences of mitochondrial genomes of *Aedes aegypti* and *Culex quinquefasciatus* and comparative analysis of mitochondrial DNA fragments inserted in the nuclear genomes. Insect Biochem Mol Biol.

[13] Harbach RE (2011). Classification within the cosmopolitan genus *Culex* (Diptera: Culicidae): the foundation for molecular systematics and phylogenetic research. Acta Trop.

[14] Liu Q, Liu X, Cirendunzhu, Woodward A, Pengcuociren, Bai L (2013). Mosquitoes established in Lhasa city, Tibet, China. Parasit Vectors.

[15] Kim NH, Lee WG, Shin EH, Roh JY, Rhee HC, Park MY (2014). Prediction forecast for *Culex tritaeniorhynchus* populations in Korea. Osong Public Health Res Perspect.

[16] Control CfD, Prevention (2013). Japanese encephalitis surveillance and immunization - Asia and the Western Pacific, 2012. MMWR.

[17] Harbach RE (2012). *Culex pipiens*: species versus species complex taxonomic history and perspective. J Am Mosquito Cont Assoc.

[18] Zou YL, Ding YR, Luo QC, Chen B (2015). The extraction method of mosquito mitochondrial genome DNA. Chin J Vector Biol Cont.

[19] Zhang NX, Zhang YJ, Yu G, Chen B (2013). Structure characteristics of the mitochondrial genomes of Diptera and design and application of universal primers for their sequencing. Acta Ento Sinica.

[20] Lowe TM, Eddy SR (1997). tRNAscan-SE: a program for improved detection of transfer RNA genes in genomic sequence. Nucleic Acids Res.

[21] Cannone JJ, Subramainian S, Schnare MN, Collett JR, Souza LM (2002). The comparative RNA web (CRW) site: an online database of comparative sequence and structure information for ribosomal, intron, and other RNAs. BMC Bioinformat.

[22] Zuker M (2003). Mfold web server for nucleic acid folding and hybridization prediction. Nucleic Acids Res.

[23] Tamura K, Peterson D, Peterson N, Stecher G, Nei M, Kumar S (2011). MEGA5: molecular evolutionary genetics analysis using maximum likelihood, evolutionary distance, and maximum parsimony methods. Mol Biol Evol.

[24] Librado P, Rozas J (2009). DnaSP v5: a software for comprehensive analysis of DNA polymorphism data. Bioinformat.

[25] Perna NT, Kocher TD (1995). Patterns of nucleotide composition at fourfold degenerate sites of animal mitochondrial genomes. J Mol Evol.

[26] Benson G (1999). Tandem repeats finder: a program to analyze DNA sequences. Nucleic Acids Res.

[27] Guindon S, Gascuel O (2003). A simple, fast, and accurate algorithm to estimate large phylogenies by maximum likelihood. Syst Biol.

[28] Posada D, Crandall KA (1998). MODELTEST: testing the model of DNA substitution. Bioinformat.

[29] Beard CB, Hamm DM, Collins FH (1993). The mitochondrial genome of the mosquito *Anopheles gambiae*: DNA sequence, genome organization, and comparisons with mitochondrial sequences of other insects. Insect Mol Biol.

[30] Mitchell SE, Cockburn AF, Seawright JA (1993). The mitochondrial genome of *Anopheles quadrimaculatus* species A: complete nucleotide sequence and gene organization. Genome.

[31] Saccone C, De GC, Gissi C, Pesole G, Reyes A (1999). Evolutionary genomics in Metazoa: the mitochondrial DNA as a model system. Gene.

[32] Krzywinski J, Li C, Morris M, Conn JE, Lima JB, Povoa MM, Wilkerson RC (2011). Analysis of the evolutionary forces shaping mitochondrial genomes of a Neotropical malaria vector complex. Mol Phylogenet Evol.

[33] Ojala D, Montoya J, Attardi G (1981). tRNA punctuation model of RNA processing in human mitochondria. Nature.

[34] Negrisolo E, Babbucci M, Patarnello T (2011). The mitochondrial genome of the ascalaphid owlfly *Libelloides macaronius* and comparative evolutionary mitochondriomics of neuropterid insects. BMC Genomics.

[35] Hong MY, Lee EM, Jo YH, Park HC, Kim SR, Hwang JS (2008). Complete nucleotide sequence and organization of the mitogenome of the silk moth *Caligula boisduvalii* (Lepidoptera: Saturniidae) and comparison with other lepidopteran insects. Gene.

[36] Chen M, Tian LL, Shi QH, Cao TW, Hao JS (2012). Complete mitogenome of the Lesser Purple Emperor *Apaturailia* (Lepidoptera: Nymphalidae: Apaturinae) and comparison with other nymphalid butterflies. Zool Res.

[37] Varani G, McClain WH (2000). The G x U wobble base pair: A fundamental building block of RNA structure crucial to RNA functions in diverse biological systems. EMBO Rep.

[38] Gutell RR, Lee JC, Cannone JJ (2002). The accuracy of ribosomal RNA comparative structure models. Nucleic Acids.

[39] Masta SE, Boore JL (2004). The complete mitochondrial genome sequence of the spider *Habronattus oregonensis* reveals rearranged and extremely truncated tRNAs. Mol Biol Evol.

[40] Miller BR, Crabtree MB, Savage HM (1996). Phylogeny of fourteen *Culex* mosquito species, including the *Culex pipiens* complex, inferred from the internal transcribed spacers of ribosomal DNA. Insect Mol Biol.

[41] Urbanelli S, Silvestrini F, Sabatinelli G, Raveloarifera F, Petrarca V, Bullini L (1995). Characterization of the *Culex pipiens* complex (Diptera: Culicidae) in Madagascar. J Med Entomol.

[42] Humeres SG, Almiron WR, Sabattini MS, Gardenal CN (1998). Estimation of genetic divergence and gene flow between *Culex pipien*s and *Culex quinquefasciatus* (Diptera: Culicidae) in Argentina. Mem Inst Oswaldo Cruz.

[43] Urbanelli S, Silvestrini F, Reisen WK, De Vito E, Bullini L (1997). Californian hybrid zone between *Culex pipiens pipiens* and *Cx. p. quinquefasciatus* revisited (Diptera: Culicidae). J Med Entomol.

[44] Smith JL, Fonseca DM (2004). Rapid assays for identification of members of the *Culex* (*Culex*) *pipiens* complex, their hybrids, and other sibling species (Diptera: Culicidae). Am J Trop Med Hyg.

[45] Cui F, Qiao CL, Shen BC, Marquine M, Raymond M (2007). Genetic differentiation of *Culex pipiens* (Diptera: Culicidae) in China. Bull Entomol Res.

