# Supplementary material for: The mitochondrial genomes of Culex tritaeniorhynchus and Culex pipiens pallens (Diptera: Culicidae) and comparison analysis with two other Culex species
Source: Parasit Vectors. 2016 Jul 21;9:406. doi: 10.1186/s13071-016-1694-z (PMC4957372; doi:10.1186/s13071-016-1694-z)
Supplement: Additional file 1: Table S1. — GenBank accession numbers for the mt genomes of the mosquito species used in this study. (PDF 21 kb) [file 13071_2016_1694_MOESM1_ESM.pdf]

Additional file 1: Table S1 The mosquito species used and their GenBank accession numbers in this study.

Species	GenBank	Species	GenBank
Culex pipiens pallens	KT851543	*Culex quinquefasciatus*	GU188856
Culex tritaeniorhynchus	KT851544	*Culex quinquefasciatus* from USA	HQ724617
Culex pipiens from Turkey	HQ724616	*Culex pipiens pipiens*	NC-015079
Culex pipiens control region	CPU69572	*Anopheles gambiae*	NC_002084
