# Supplementary material for: The mitochondrial genomes of Culex tritaeniorhynchus and Culex pipiens pallens (Diptera: Culicidae) and comparison analysis with two other Culex species
Source: Parasit Vectors. 2016 Jul 21;9:406. doi: 10.1186/s13071-016-1694-z (PMC4957372; doi:10.1186/s13071-016-1694-z)
Supplement: Additional file 2: Figure S1. — Predicted secondary structure for 22 tRNAs (a), 12S rRNA (b) and 16S rRNA (c) in Cx pipiens pallens and Cx tritaeniorhynchus mt genomes. (PDF 240 kb) [file 13071_2016_1694_MOESM2_ESM.pdf]

Additional file 2: Figure S1 Predicted secondary structures for 22 tRNAs (a), 12S rRNA (b) and 16S rRNA (c) in *Cx pipiens pallens* and *Cx tritaeniorhynchus* mtgenomes. Bracketed, crossed, and underlined amino acids were substituted, deleted and inserted in the mtgenome of *Cx pipiens pallens* comparison with the *Cx tritaeniorhynchus*, respectively. The dashes indicate Watson-Crick bonds, and red pairs indicate mistaken bonds. H: helices; I: interior loop; T: terminal loop.

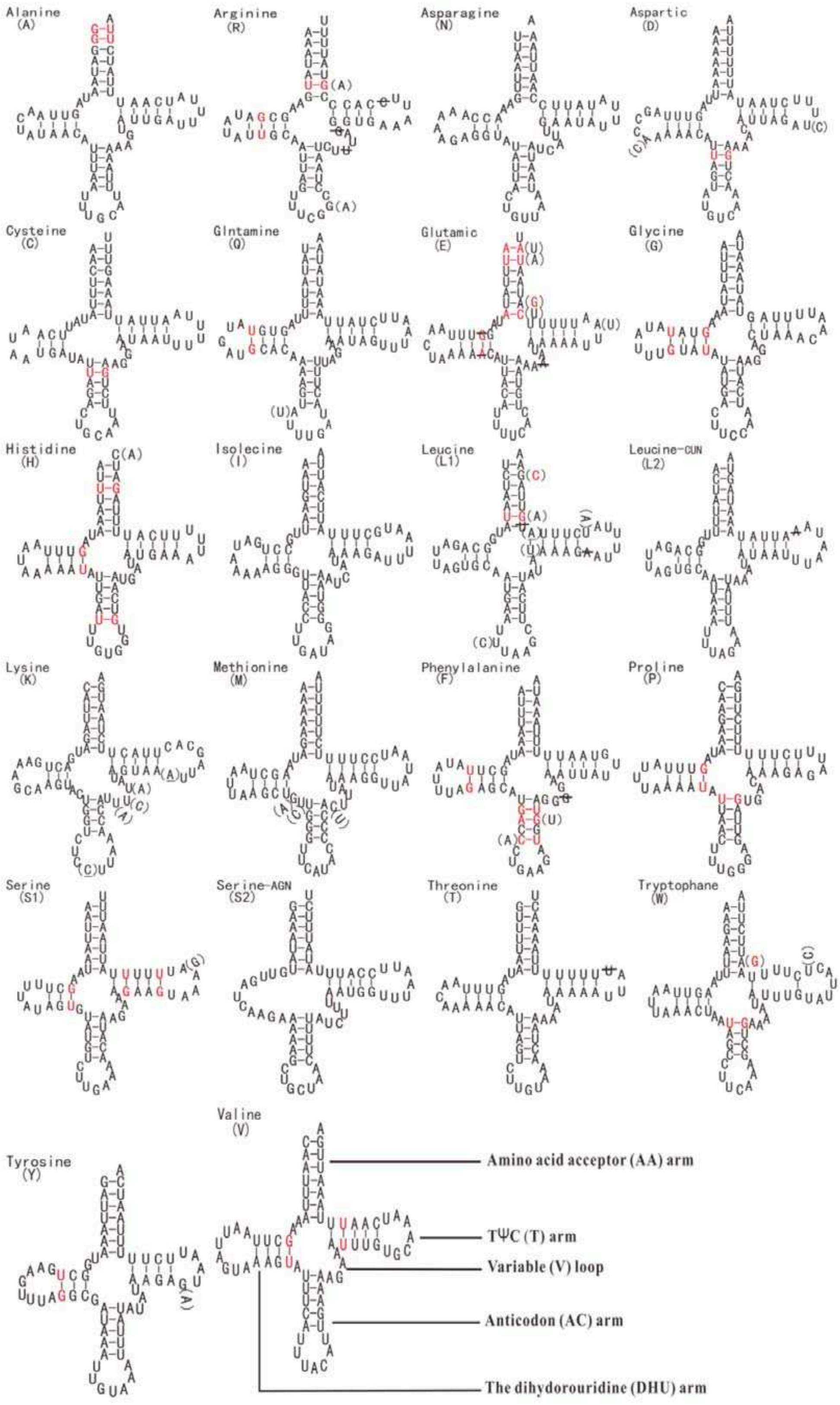
Alanine (A)
Arginine (R)
Asparagine (N)
Aspartic (D)
Cysteine (C)
Glntamine (Q)
Glutamic (E)
Glycine (G)
Histidine (H)
Isolecine (I)
Leucine (L1)
Leucine-CUN (L2)
Lysine (K)
Methionine (M)
Phenylalanine (F)
Proline (P)
Serine (S1)
Serine-AGN (S2)
Threonine (T)
Tryptophane (W)
Tyrosine (Y)
Valine (V)
Amino acid acceptor (AA) arm
TΨC (T) arm
Variable (V) loop
Anticodon (AC) arm
The dihydorouridine (DHU) arm

a

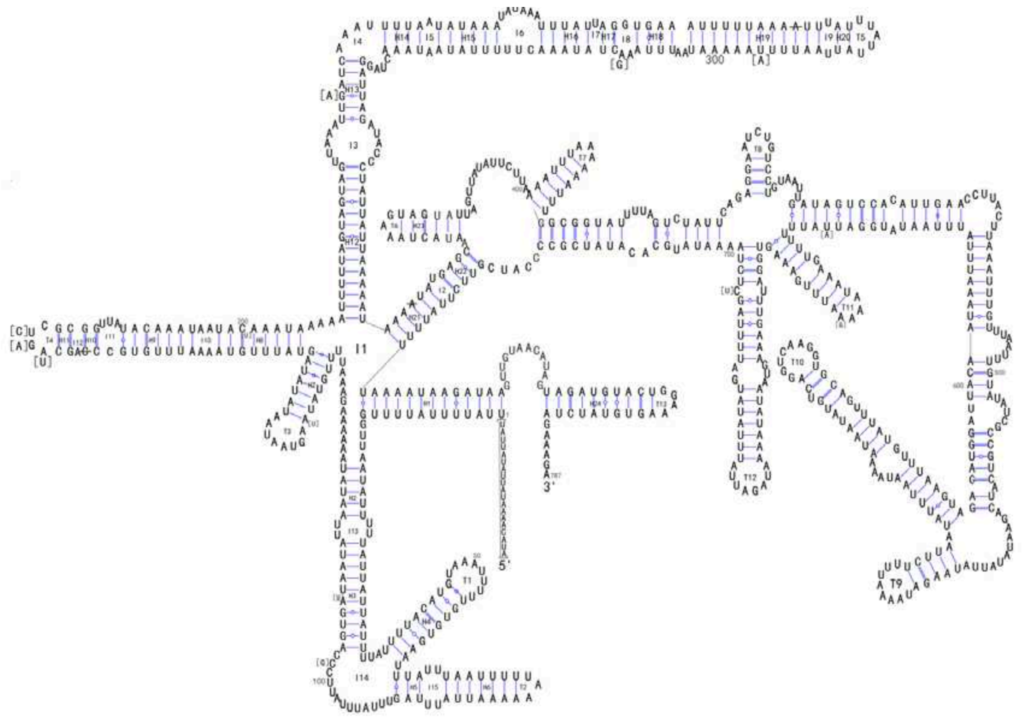

b

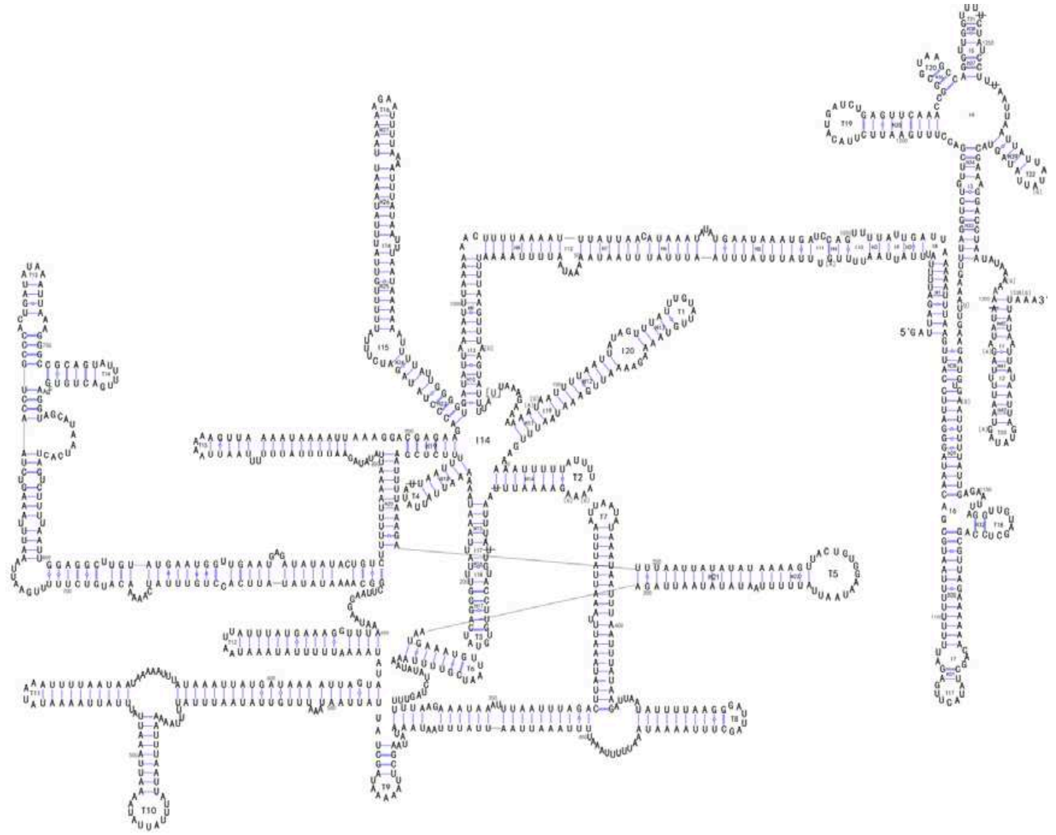

C
